# Analysis of Lumbar Sagittal Curvature in Spinal Decompression and Fusion for Lumbar Spinal Stenosis Patients under Roussouly Classification

**DOI:** 10.1155/2020/8078641

**Published:** 2020-05-01

**Authors:** Guoqiang Zhang, Yong Yang, Yong Hai, Jinjun Li, Xuehu Xie, Shitong Feng

**Affiliations:** ^1^Department of Orthopedics, Beijing Friendship Hospital, Capital Medical University, Beijing 100050, China; ^2^Department of Orthopedics, Beijing Chao-Yang Hospital, Capital Medical University, Beijing 100020, China

## Abstract

To evaluate the clinical significance of spinal decompression and fusion for lumbar spinal stenosis in old patients under Roussouly classification, 160 old patients (>60 year old) with lumbar spinal stenosis underwent spinal decompression, and fusion were retrospectively studied. According to Roussouly classification, patients were divided into 4 groups, in which Roussouly types I, II, and IV were the nonstandard group and Roussouly type III was the standard group. Visual analog scale (waist, leg) and Oswestry disability index (ODI) scores were recorded before operation and at the final follow-up. All patients improved the sagittal curvature: for patients in Roussouly types I and II, there were statistically significant differences in terms of postoperative global lordosis (GL), global kyphosis (GK), sacral slope (SS), sagittal vertical axis (SVA), and pelvic tilt (PT) compared with that before surgery (all *P* < 0.001); patients in Roussouly type IV obtained similar results with type III after surgery. The four groups showed significant improvement in ODI and VAS scores at final follow-up (all *P* < 0.001). After regrouping at the final follow-up, the proportion of the standard type (Roussouly type III) patients was increased compared with preoperative. In conclusion, Roussouly classification has important guiding significance in spinal decompression and fusion for old patients (>60 years) with lumbar spinal stenosis.

## 1. Introduction

The spine-pelvis plays an important role in maintaining the upright posture of the human body [1, 2]. The spino-pelvic sagittal balance allows the body to maintain an upright posture with minimal energy consumption, while cushioning the impact and shock of movement on the spinal cord [3, 4]. Human beings have adjacent physiological curvature, including cervical kyphosis, thoracic kyphosis, lumbar kyphosis, and sacral kyphosis. In this spine-pelvis-hinged structure, adjacent kyphosis and kyphosis segments are closely related to each other. Lumbar lordosis plays a bridging role between the pelvis and the thoracic curvature in the sagittal sequence and is the core of the adjustment of sagittal spino-pelvic and balance [5].

Lumbar degenerative disease, including lumbar spinal stenosis disease, lumbar intervertebral disc protrusion, and lumbar olisthe disease, often accompanied by pathological changes, resulting in lumbocrural pain and neural dysfunction, serious and even completely lose normal life ability and high morbidity [6, 7]. With the progress of lumbar degeneration, a series of pathological changes, such as narrowing of vertebral space, instability of facet joints, and gradual decreases of lumbar lordosis, could lead to the spino-pelvic sagittal imbalance [8]. Spino-pelvic sagittal imbalance patients often accompanied by intractable back pain and fatigue, upper body forward, and even difficulty looking straight at the eye [9]. Numerous studies have reported that restoring and maintaining the spino-pelvic sagittal balance in the treatment of degenerative diseases of the spine is crucial for the improvement of surgical efficacy and patients' quality of life [3, 10].

The current gold standard treatment for degenerative spinal diseases is spinal fusion [11]. With the increase of life expectancy, a growing number of patients was treated with cervical spine fusion surgery due to radiculopathy or myelopathy resulting from lumbar degenerative disease or spino-pelvic sagittal imbalance [12]. The indications for spine fusion surgery were the following: nonsurgical treatment of uncontrolled and intolerable lower limb pain with or without lower back pain; persistent lower limb symptoms and progressive intermittent claudication which had no significant effect after 2-3 months of nonsurgical treatment; severe nerve compression and progressive loss of nerve function; and cauda equina syndrome patients with consistent symptoms and signs, and imaging examination should be considered for surgical treatment [13, 14].

Roussouly et al. classified the sagittal alignment of human in a standing position into four types according to spinal and pelvic parameters [15]. However, the correlation between the lumbar-pelvic parameters and spino-pelvic sagittal balance was still unclearly elucidated. Roussouly types may provide an objective way to explore such relationship after posterior lumbar spinal decompression and interbody fusion since their preoperative connections were interpreted in previous literature [16]. Therefore, under Roussouly classification, this study aim to evaluate the change of the lumbar-pelvic parameters and spino-pelvic sagittal balance, and the clinical significance of spinal decompression and fusion for lumbar degenerative diseases patients.

## 2. Materials and Methods

### 2.1. Patients

A total of 160 patients with lumbar spinal stenosis (73 males and 87 females, average age: 67.38 ± 4.63 years) underwent posterior lumbar spinal decompression and pedicle screw internal fixation bone grafting and fusion at our hospital from January 2014 to December 2015 and were retrospectively follow-up studied by full-length and spine lateral X-rays (follow-up: 15-22 months). This study was approved by the Institutional Review Board of Beijing Friendship Hospital, Capital Medical University. Informed consent was obtained from all patients. The inclusion criteria of patients were as follows: (1) aged > 60 years old; (2) with lower back pain; (3) with at least 3 months of ineffective conservative treatment before surgery; and (4) pre and postoperative full-length and spine lateral X-rays of the spine were available. The exclusion criteria of patients were as follows: (1) had previous history of lumbar internal fixation; (2) had spinal and pelvic deformity and lumbar fracture and fracture nonunion; (3) had metabolic bone disease, lumbar spinal canal tumors and space-occupying spinal cord diseases, infectious diseases of the lumbar spine, severe hip and knee diseases, and other degenerative diseases of the lumbar spine, such as simple lumbar disc herniation and lumbar spondylolisthesis; (4) with severe internal diseases and surgical contraindications; and (5) mentally ill, unable to cooperate.

### 2.2. Radiological Parameters and Roussouly Classification

**Figure 1:**
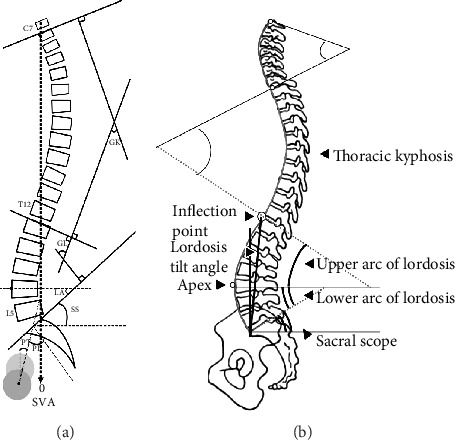
(a, b) Sagittal parameters of the spine under Roussouly classification: sagittal parameters of lumbar spine: inflection point (IP), lordosis tilt angle (LTA), apex (A), global lordosis (GL), lower arc (LA), and upper arc (UA); pelvic incidence (PI), pelvic tilt (PT), and sacral slope (SS); sagittal vertical axis (SVA); global kyphosis (GK).

Before surgery and at the final follow-up, the patients who met the inclusion criteria were examined by full-length and spine lateral X-rays, with both shoulders bent forward for 30 during the radiography to ensure the most natural state of lumbar lordosis. The radiological parameters included (1) sagittal parameters of lumbar spine: inflection point (IP), lordosis tilt angle (LTA), apex (A), global lordosis (GL), lower arc (LA), and upper arc (UA); (2) pelvic incidence (PI), pelvic tilt (PT), and sacral slope (SS); (3) sagittal vertical axis (SVA); and (4) global kyphosis (GK) (Figure 1).

**Figure 2:**
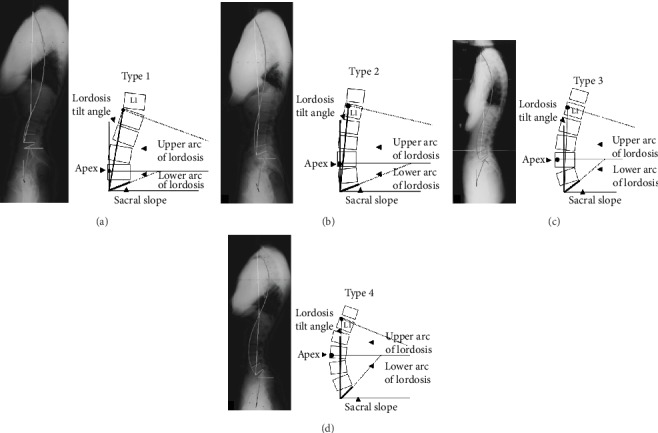
Roussouly classification. A four-part classification of morphology was used to classify each patient (a–d).

All patients were categorized under Roussouly morphological classification according to their preoperative PI, SS, thoracic, and lumbar alignments [15]. To avoid intraobserver bias, all radiographs were reviewed by two senior spine surgeons, respectively. If they disagreed, a third one was invited to make a final decision.  Figure 2 shows a detailed Roussouly morphological classification method. The detailed descriptions of Roussouly types I-IV were accorded to previous study [12]. In a clinical study, according to Roussouly classification, patients were divided into the nonstandard group (Roussouly types I, II, and IV) and the standard group (Roussouly type III) [15]. The imaging software (UniWeb; Shanghai Daijia medical Information System Co., Ltd., Shanghai, China) was used to design the lumbar curvature that needed to be adjusted for patients in the nonstandard group, such as the height of the intervertebral fusion device and the length and the degree of prebending of the screw rod, so as to make quantitative preparation for the improvement of lumbar curvature.

### 2.3. Surgical Procedure

The patient were in a prone position with the lumbar lordosis and the abdomen suspended. Posterior midline incision (6-12 cm) was determined according to the fusion segment and scope. The paravertebral muscles were detached along the periosteum of both sides of the spinous process to the lateral side of the bilateral facet joints, and pedicle screws were placed at corresponding segments. According to symptoms and radiographic features, after confirming the affected segments, the vertebral plate and facet joints were exposed, the articular process and part of the lamina were resected, and the proliferous hypertrophy of yellow ligament was removed to fully exposure of the vertebral posterior wall. The nerve root was pulled into the inside to expose the intervertebral disc for resection. Then, bone grafting was performed in intervertebral space, mold was tested and Cage with appropriate bone filling was placed, and pressurized forceps was placed on the connecting rod to restore normal physiological lumbar lordosis. All patients recieved postoperative negative pressure drainage for 24-48 hours, lie in bed and wear waist to exercise 2-3 weeks.

### 2.4. Clinical Evaluations

Preoperative and follow-up whole-spine radiographs in the standing position were obtained preoperatively at 3 months, 6 months, 12 months, 24 months, and the final follow-up months after surgery. All the patients were asked to complete the Oswestry Disability Index (ODI) for the VAS for back pain and leg pain at preoperative and at final follow-up. The VAS for pain intensity ranged from 0 to 10, the ODI score ranged from 0 to 50 [17, 18].

### 2.5. Statistical Analysis

Data were analyzed using statistical software (SPSS 20.0; SPSS Inc., USA). According to Roussouly classification, patients were divided into 4 groups to understand the changes in the number of patients before and after surgery. Paired *t* test was used to analysis the radiological parameters and functional scores in the four groups before surgery and at the final follow-up. Significance was set at *P* < 0.05.

## 3. Results

Demographic data of the enrolled patients were shown in Table 1. The patients were divided into 4 groups with Roussouly classification, and intergroup comparisons of preoperative or postoperative factors revealed that there was no significant difference among groups including age, gender, BMI, duration of symptom, number of fusion segments, operative time, blood loss, length of hospital stay, or follow-up time.

The comparisons of whole spinal sagittal parameters of all subjects in different Roussouly types between pre and postoperation were shown in Tables 2 and 3. For patients in Roussouly types I and II, compared with preoperative, there were statistically significant differences in terms of postoperative GL, GK, SS, SVA, and PT (all *P* < 0.001), while PI had no significant difference. For patients in Roussouly types III and IV, compared with preoperative, there was no statistically significant difference in terms of postoperative GL, GK, and PI, while SVA and SS had significant difference (all *P* < 0.001). All the results showed that the improvement of lumbar curvature in patients, especially patients in Roussouly type I, Roussouly type II, and Roussouly type IV groups.

The four groups showed significant improvement from baseline in ODI scores, VAS scores for waist and leg pain at the final follow-up time (all *P* < 0.001) (Table 4). For all patients, preoperative and postoperative change of VAS between waist and leg had statistically significant difference (waist vs. leg: 4.58 ± 1.88 vs. 2.96 ± 1.53, *P* < 0.001), which indicated that the postoperative functional scores were improved.

**Figure 3:**
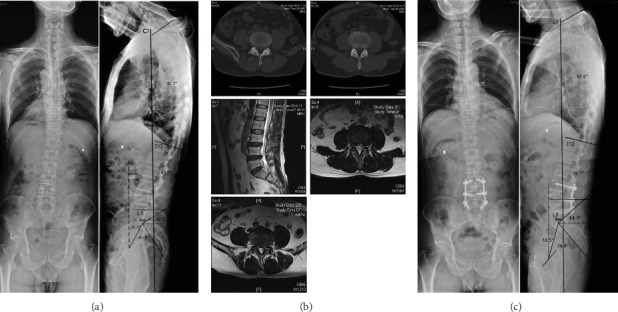
A 60-year-old male with diagnosed with lumbar spinal stenosis and had back pain for 4 years, aggravating pain in both lower limbs for 6 months. (a) Preoperative X-ray showed SVA = 20.4 mm, SS = 32.7, Roussouly type I, with the apex of lordosis at L5 upper edge, GL = 47.7°, PI = 60.8°, PT = 26.6°, SS = 32.7°, GK = 40.7°. (b) Preoperative lumbar CT and MRI showed L3/4, L4/5 segment disc herniation, facet joint hyperplasia and cohesion, and dural compression. (c) Postoperative X-ray showed that SVA = 0 mm, SS = 44.0°, Roussouly type III, the lumbar lordosis vertex was at the L4 midpoint, GL = 55.7°, PI = 58.8°, PT = 16.5°, SS = 44.0°, and GK = 50.0°. SS, GL, and GK increased; SVA and PT decreased; PI unchanged. Lordosis vertex moved up to the L4 midpoint, classification from Roussouly types I to III (standard).

**Figure 4:**
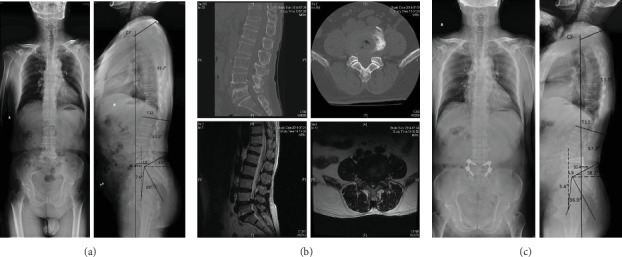
A 67-year-old male with diagnosed with lumbar spinal stenosis and had back pain for 3 years, with intermittent claudication. (a) Preoperative X-ray showed SVA = 46.3 mm, SS = 32, Roussouly type II, lumbar lordosis apex at L4 base, GL = 42.1°, PI = 39.0°, PT = 7.6°, SS = 32.0°, GK = 32.2°. (b) Preoperative lumbar CT and MRI showed L4/5 segment disc herniation, yellow ligament hypertrophic, dural compression, and spinal canal narrowing. (c) Postoperative X-ray showed that SVA = 10.4 mm, SS = 38.2°, Roussouly type III, the lumbar lordosis vertex was at the L4 midpoint, GL = 57.2°, PI = 5.4°, PT = 36.9°, SS = 38.2°, GK = 53.5°. SS, GL, and GK increased; SVA and PT decreased; PI unchanged. Lordosis vertex moved up to the L4 midpoint, classification from Roussouly types II to III (standard).

Patients were reclassified according to Roussouly classification at final follow-up. There were statistical differences in term of number case in the different Roussouly types at preoperation and at final follow-up (*P* < 0.001). The specific manifestation was that the number of type I and type II patients decreased with statistical differences and the number of type IV patients decreased with no statistical differences at final follow-up compared with that of preoperative; while the number of type III patients increased with statistical differences at the final follow-up compared with that of preoperative. Meanwhile, there were statistical differences in terms of patients' number between the nonstandard group and standard group before and after operation (*P* < 0.001), indicating the proportion of adjusted group patients increased at the final follow-up compared with that before surgery (Table 5). Figures 3 and 4 show the two typical cases, both changed to Roussouly type III.

## 4. Discussion

Before spinal fusion, patients with degenerative diseases of lumbar spine were examined by full-length and spine lateral X-rays to measure the parameters of sagittal spine-pelvis, and the corrective angle of lumbar lordosis, especially lower lumbar lordosis, was predicted preoperatively according to the size of SS. In this way, not only thorough decompression and relief of nerve compression during operation but also recovery of lumbar lordosis and spino-pelvic sagittal balance and prevention of spinal instability and muscle fatigue caused by spino-pelvic sagittal imbalance after operation, so as to improve the clinical effect of spinal fusion and avoid the second orthopaedic operation [19].

In 2017, Sebaaly et al. proposed the classification of the degenerative spine and its possible outcome based on Roussouly classification, which was applicable to the classification of normal people and would help orthopedic surgeons to distinguish patients' initial spinal classification and restore it to the desired curvature [20]. There was no report in China about using lumbar curvature parameters of Roussouly classification to evaluate the recovery of lumbar sagittal balance and the correlation between surgical efficacies in patients with lumbar degenerative diseases. By studying the sagittal plane parameters of lumbar spine, the reconstruction of physiological curvature of lumbar spine during operation can be guided to improve the curative effect, relieve symptoms better, and further improve the postoperative quality of life of patients [21]. Preoperative measurement of patients' lumbar sagittal plane parameters of Roussouly classification can guide the selection of surgical procedures and maximize the recovery of patients' lumbar physiological curvature [22].

In this study, according to Roussouly Classification, the imaging software was used to design the lumbar curvature that needed to be adjusted for patients in the nonstandard group. It was found that the height of intervertebral space and the LA of Roussouly types I, II, and IV patients were restored after Cage implantation during surgical decompression. The lower LA occupied an important proportion in the GL, which was equal to the SS, so SS was restored at the same time. With long-term follow-up, for Roussouly types I and II patients, the full-length and lateral spine X-rays showed that the effective GK was recovered compared with that of before surgery, and the distance of the C7 plumb line from the SVA was close to or within the normal range (0-50 mm). However, PI is a constant anatomical parameter, PT decreased due to the increase of SS. For Roussouly type IV patients with larger SS and hypercurvature coordination of lumbar before surgery, the SS, GL, GK, SVA, PI, and PT of the patients obtained similar results with type III after surgery, indicating that the sagittal position of the spine had reached balance.

Roussouly classification provides a good approach and clinical strategy for clinical surgeons. In 2019, Sebaaly et al. [23] and Pizones et al. [24] studied the application of Roussouly classification in adult spinal deformity. The two experts simultaneously proposed that the reverted Roussouly standard type (type III) could significantly reduce the occurrence of mechanically related complications, which was nearly three times lower than that of patients who did not recover to the standard type. All indicated the importance of restoring Roussouly standard type, which was coincided with the concept and direction of our study. In this study, Roussouly classification was used to observe the patient number change and the functional score of different Roussouly types at preoperative and final follow-up. The result indicated patients who returned to the standard type not only improved the sagittal curvature but also improved the functional score. Of course, not all patients in the nonstandard type could recover into the standard type after operation, some patients with postoperative Roussouly type did not change and might still show improvement in symptoms and function. Achieving complete asymptomatic and spinal balance is a goal pursued by clinical surgeons but not dogmatically forcing all patients change to Roussouly type III. All the results fully demonstrated that patients with lumbar spinal stenosis not only need decompression of the spinal canal and nerve root release but also restoration of spino-pelvic sagittal balance. Roussouly classification perhaps is not perfect and not be consistent across all surgeons, but it was tried to test the efficacy of the surgery. We believe that as more patients are included and methodology improves, we will gradually improve this evaluation method in future studies.

This study has several limitations. Firstly, it is a retrospective study, patients were old (>60 years), and the number of patients in different Roussouly types was relatively small, biases may occur. Secondly, there was no specific statistical analysis of the association between the parameters of spino-pelvic and the patient's clinical score. Thirdly, because of the small sample size, it is not possible to classify the effect of spinal fusion on long and short segments. Fourthly, due to the relatively short follow-up period, it is not possible to analyze the long-term effects of surgery on lumbar curvature parameters and other sagittal parameters, and the effects of lumbar curvature correction on adjacent segment degeneration. In the future, we will collect larger sample size to study the relationship between lumbar curvature loss and clinical score changes after spinal fusion, and the temporal relationship in the change of Roussouly types and the various sagittal parameters at different follow-up time points.

## 5. Conclusion

Roussouly classification has important guiding significance in spinal decompression and fusion for old patients with (>60 years old) lumbar spinal stenosis.

## Figures and Tables

**Table 1:** Baseline characteristics of the 4 groups with Roussouly classification.

	Total	Roussouly type I	Roussouly type II	Roussouly type III	Roussouly type IV	*P* value
Age (years)	67.38 ± 4.63	60.49 ± 4.39	68.03 ± 4.54	66.29 ± 5.65	64.47 ± 3.46	0.033
Gender (n)						0.66
Male	73	16	42	6	9	
Female	87	23	47	11	6	
BMI (kg/m^2^)	23.13 ± 2.61	23.92 ± 2.85	23.03 ± 2.52	23.59 ± 2.53	23.73 ± 2.74	0.644
Duration of symptom (months)	19.71 ± 8.47	20.56 ± 7.42	19.29 ± 8.63	18.59 ± 8.28	21.27 ± 10.54	0.705
Number of fusion segments	3.06 ± 1.37	3.36 ± 1.37	2.99 ± 1.38	2.65 ± 1.32	3.13 ± 1.30	0.297
Operative time (minutes)	163.25 ± 55.90	159.64 ± 55.15	163.22 ± 55.90	173.65 ± 49.35	161.00 ± 68.08	0.858
Blood loss (ml)	271.00 ± 111.67	306.15 ± 107.43	265.62 ± 108.63	241.76 ± 120.99	244.67 ± 118.25	0.107
Length of hospital stay (days)	13.73 ± 3.24	14.13 ± 3.41	13.56 ± 3.19	13.41 ± 2.76	14.07 ± 3.8	0.767
Follow-up (months)	18.49 ± 2.33	18.21 ± 2.09	18.38 ± 2.23	18.82 ± 2.60	19.47 ± 1.99	0.250

**Table 2:** Statistical analysis of pre and postoperative parameters (GL, GK, and SVA) of patients in four types.

Roussouly classification	GL (°)		GK (°)		SVA (mm)	
	Preoperative	Postoperative	Preoperative	Postoperative	Preoperative	Postoperative
Type I	26.55 ± 4.60	31.08 ± 4.64	34.40 ± 6.82	39.46 ± 6.82	52.08 ± 13.14	40.34 ± 13.13
t value	-104.009		-108.538		270.535	
P value	<0.001		<0.001		<0.001	
Type II	38.47 ± 4.73	42.13 ± 4.83	35.37 ± 6.17	39.78 ± 6.16	40.82 ± 11.78	31.49 ± 11.26
t value	-47.274		-67.479		52.904	
P value	<0.001		<0.001		<0.001	
Type III	39.51 ± 4.29	40.69 ± 4.24	41.18 ± 5.44	41.87 ± 5.04	40.01 ± 6.22	31.84 ± 6.23
t value	-1.769		-1.015		111.426	
P value	0.096		0.325		<0.001	
Type IV	54.45 ± 6.89	53.77 ± 5.81	40.08 ± 4.31	43.16 ± 3.49	30.07 ± 8.57	32.17 ± 8.53
t value	1.225		-1.844		-33.120	
P value	0.241		0.086		<0.001	

GL: global lordosis; GK; global kyphosis; SVA: sagittal vertical axis.

**Table 3:** Statistical analysis of pre- and post-operative parameters (PI, PT, and SS) of patients in four types.

Roussouly classification	PI (°)		PT (°)		SS (°)	
	Preoperative	Postoperative	Preoperative	Postoperative	Preoperative	Postoperative
Type I	45.70 ± 5.59	46.44 ± 8.37	23.97 ± 4.00	16.93 ± 4.01	19.06 ± 5.76	33.46 ± 6.64
t value	-0.558		142.830		-10.796	
P value	0.580		<0.001		<0.001	
Type II	47.92 ± 4.18	59.63 ± 4.44	20.46 ± 3.29	16.45 ± 3.26	28.38 ± 3.61	35.36 ± 5.65
t value	-1.500		124.418		-11.134	
P value	0.137		<0.001		<0.001	
Type III	61.13 ± 4.90	61.85 ± 4.20	18.73 ± 3.67	18.22 ± 3.84	37.75 ± 1.47	39.33 ± 1.47
t value	-0.794		1.140		-26.882	
P value	0.439		0.271		<0.001	
Type IV	62.06 ± 3.14	60.50 ± 4.07	14.67 ± 2.53	15.50 ± 2.58	48.96 ± 1.99	45.02 ± 3.10
t value	1.604		-11.155		6.472	
P value	0.131		<0.001		<0.001	

PI; pelvic incidence; PT: pelvic tilt; SS: sacral slope.

**Table 4:** The functional changes before and at the final follow-up of thoracic and lumbar of patients in four types.

	Roussouly type I	Roussouly type II	Roussouly type III	Roussouly type IV
VAS score (leg)				
Preoperative	5.28 ± 1.12	5.42 ± 1.09	5.59 ± 1.06	5.07 ± 1.03
Final follow-up	2.62 ± 1.21	2.38 ± 1.07	2.24 ± 1.25	2.20 ± 0.86
P value	<0.001	<0.001	<0.001	<0.001
VAS score (waist)				
Preoperative	7.26 ± 1.43	6.96 ± 1.42	6.41 ± 1.50	7.40 ± 1.24
Final follow-up	2.46 ± 1.19	2.30 ± 1.11	2.47 ± 1.18	3.07 ± 1.03
P value	<0.001	<0.001	<0.001	<0.001
ODI score				
Preoperative	37.85 ± 3.67	37.98 ± 3.64	38.18 ± 3.00	38.47 ± 3.83
Final follow-up	13.23 ± 2.97	13.27 ± 2.91	13.06 ± 2.59	12.13 ± 2.59
P value	<0.001	<0.001	<0.001	<0.001

VAS: visual analog scale; ODI: Oswestry Disability Index.

**Table 5:** The changes number of patients preoperation and at final follow-up according to Roussouly classification (*n* = 160).

	Preoperation	Final follow-up	*χ* ^2^	*P*
Roussouly classification	Number of case (*n*, %)	Number of case (*n*, %)	135.818	<0.001
Roussouly type I	39 (24.4%)	17 (10.6%)		
Roussouly type II	89 (55.6%)	44 (27.5%)		
Roussouly type III	17 (10.6%)	87 (54.4%)		
Roussouly type IV	15 (9.4%)	12 (7.5%)		
Types			15.960	<0.001
Standard group	17 (10.6%)	87 (54.4%)		
Nonstandard group	143 (89.4%)	73 (45.6%)		

The Roussouly type III is defined as standard, the other three types (Roussouly type II, Roussouly type III, and Roussouly type IV) are defined as nonstandard.

## Data Availability

The data used to support the findings of this study are available from the corresponding author upon request.
